# Effects of a recombinant gene expression on ColE1-like plasmid segregation in *Escherichia coli*

**DOI:** 10.1186/1472-6750-11-18

**Published:** 2011-03-01

**Authors:** Mladen Popov, Stefan Petrov, Genoveva Nacheva, Ivan Ivanov, Udo Reichl

**Affiliations:** 1 Institute of Molecular Biology "Roumen Tsanev", Bulgarian Academy of Sciences, Acad. G. Bonchev Str., 21, 1113 Sofia, Bulgaria; 2 Max Planck Institute for Dynamics of Complex Technical Systems, Sandtorstr.1, 39106 Magdeburg, Germany

## Abstract

**Background:**

Segregation of expression plasmids leads to loss of recombinant DNA from transformed bacterial cells due to the irregular distribution of plasmids between the daughter cells during cell division. Under non-selective conditions this segregational instability results in a heterogeneous population of cells, where the non-productive plasmid-free cells overgrow the plasmid-bearing cells thus decreasing the yield of recombinant protein. Amongst the factors affecting segregational plasmid instability are: the plasmid design, plasmid copy-number, host cell genotype, fermentation conditions etc. This study aims to investigate the influence of transcription and translation on the segregation of recombinant plasmids designed for constitutive gene expression in *Escherichia coli *LE392 at glucose-limited continuous cultivation. To this end a series of pBR322-based plasmids carrying a synthetic human interferon-gamma (hIFNγ) gene placed under the control of different regulatory elements (promoter and ribosome-binding sites) were used as a model.

**Results:**

Bacterial growth and product formation kinetics of transformed *E. coli *LE392 cells cultivated continuously were described by a structured kinetic model proposed by Lee et al. (1985). The obtained results demonstrated that both transcription and translation efficiency strongly affected plasmid segregation. The segregation of plasmid having a deleted promoter did not exceed 5% after 190 h of cultivation. The observed high plasmid stability was not related with an increase in the plasmid copy-number. A reverse correlation between the yield of recombinant protein (as modulated by using different ribosome binding sites) and segregational plasmid stability (determined by the above model) was also observed.

**Conclusions:**

Switching-off transcription of the hIFNγ gene has a stabilising effect on ColE1-like plasmids against segregation, which is not associated with an increase in the plasmid copy-number. The increased constitutive gene expression has a negative effect on segregational plasmid stability. A kinetic model proposed by Lee et al. (1985) was appropriate for description of *E. coli *cell growth and recombinant product formation in chemostat cultivations.

## Background

Plasmid segregation is a well known phenomenon in recombinant DNA biotechnology and major factor reducing the yield of recombinant proteins [1-3]. The loss of multicopy plasmids is thought to be due to the irregular distribution of plasmids between the daughter cells during cell division [4]. This results in heterogeneous cell populations in which the plasmid-free cells overgrow the plasmid-bearing cells under non-selective conditions [5,6]. Eventually, the overall plasmid content in the populations, and therefore the productivity of cultivations decreases. The imposition of plasmid-free over plasmid-bearing cells depends on the difference between their specific growth rates and on the generation rate of plasmid-free cells [5,7,8]. These characteristics can be affected by a large number of factors such as mechanism and rate of plasmid replication, plasmid copy-number [9], plasmid multimerization [4,10] eventually leading to the so called dimer/multimer catastrophe [11,12], concatameric replication [13], presence of partitioning elements [4], cultivation conditions (temperature [14], pH [15], composition of growth medium [15-18], dilution rate [19], agitation rate [20]), host cell genotype [21], etc.

Vectors for bacterial gene expression are usually multicopy plasmids with a relaxed control of replication, carrying a strong promoter and a strong ribosome binding site. It has been observed that the extensive gene expression thus achieved causes significant reduction in the specific growth rate of plasmid-harbouring cells (mainly due to metabolic burden) [22-25].

Taking into consideration that gene expression includes two consecutive steps, transcription and translation, and also that in prokaryotes these two processes are not separated in space and time, one can assume that each stage interferes with plasmid replication and therefore with its segregation. It was shown for instance that extensive transcription causes a reduction in plasmid copy-number and favours plasmid segregation [26-28].

The aim of this work is to investigate the segregation of a series of pBR322-based plasmids expressing with different efficiency human interferon-gamma (hIFNγ) in *E. coli *LE392 cells.

The hIFNγ is a cytokine endowed with multiple biological activities [29]. It is secreted in the human fluids in minute amounts and due to this it has been targeted by the recombinant DNA technology in the early 80's. In this study the hIFNγ was chosen as a model because of its importance for the pharmaceutical industry and medicine.

In the investigated plasmids the hIFNγ gene was placed under the control of different regulatory elements (promoter and ribosome-binding sites) in order to vary the efficiency/level of both transcription and translation. To evaluate the impact of these processes on plasmid segregation the models of Stewart & Levin [30] and Lee et al. [31] were employed.

## Methods

### Plasmids and Bacterial Strain

**Figure 1:**
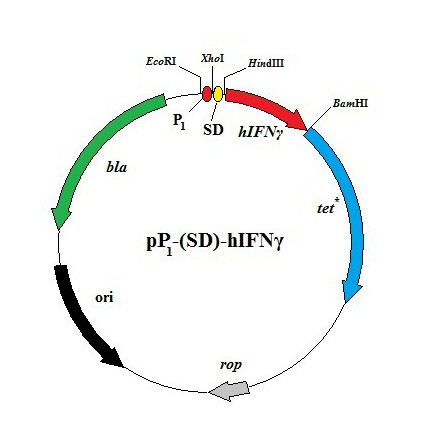
**Functional map of the plasmid pP_1_-(SD)-hIFNγ**. P_1 _- strong constitutive promoter; SD - Shine & Dalgarno consensus sequence; *hIFNγ *- human interferon gamma gene coding for 143 amino acids; *bla *(Ap^R^) - β-lactamase gene; tet*- truncated tetracycline resistance gene; *rop *- gene coding for the ROP protein (regulating plasmid copy-number); ori - origin of replication.

The plasmids used are listed in Table 1. The plasmid pP_1_-(SD)-hIFNγ (Figure 1) is a derivative of pBR322 where the fragment between *Eco*RI and *Bam*HI restriction sites (the latter located in the *tet *(Tc^R^) gene) is replaced by a cassette containing a strong constitutive promoter P_1 _(analogue of the T5 bacteriophage early promoter), a ribosome-binding site (the consensus Shine & Dalgarno sequence AAGGAGGT) and a hIFNγ gene. The mRNA transcribed from the P_1 _promoter is dicistronic and consists of the complete hIFNγ sequence plus a part of the *tet *gene (downstream of the *Bam*HI site). A translation stop codon TAA is introduced after the last codon of the hIFNγ gene. The plasmids pΔP_1_-(ΔSD)-hIFNγ, pP_1_-(ΔSD)-hIFNγ and pP_1_-(4SD)-hIFNγ are derivatives of the plasmid pP_1_-(SD)-hIFNγ. The construct pΔP_1_-(ΔSD)-hIFNγ was derived from pP_1_-(SD)-hIFNγ by removing the *Eco*RI/*Hin*dIII fragment (bearing both the P_1 _promoter and the SD sequence), blunting and ligation of the rest of the plasmid. The plasmid pP_1_-(ΔSD)-hIFNγ was constructed by removing the SD sequence from the plasmid pP_1_-(SD)-hIFNγ (by *Xho*I and *Hin*dIII), blunting and ligation. The construct pP_1_-(4SD)-hIFNγ is derivative of the plasmid pP_1_-(SD)-hIFNγ in which a cluster of four tandemly repeated SD sequences (AAGGAGGTTTAACGTAAGGAGGTTTATCGAGAAGGAGGTTTAACGTAAGGAGGT, where the consensus SD sequence is underlined) was substituted for the single SD sequence. As seen from the map (Figure 1), all plasmids have the phenotype Ap^R^,Tc^S^. The plasmid pGEM-BD used for the QPCR is described elsewhere [32].

**Table 1:** Plasmid constructs.

Plasmid	Properties	
	
	**Promoter P**_**1**_	Ribosome-binding site
pP_1_-(SD)-hIFNγ	(+)	(+) (1 × SD)

pΔP_1_-(ΔSD)-hIFNγ	(-)	(-)

pP_1_-(ΔSD)-hIFNγ	(+)	(-)

pP_1_-(4SD)-hIFNγ	(+)	(+) (4 × SD)

The *E. coli *strain LE392 (*supE*44 *supF*58 *hsdR*514 *galK*2 *galT*22 *metB*1 *trpR*55 lac*Y*1) [28] was used as a host strain throughout this work.

### Media

LB (Luria-Bertani) medium [28] was used for preparation of competent cells and in the plasmid stability assay.

M9 minimal medium [28] supplemented with trace element stock solution (1:1000), glucose (final concentration 1.96 g/L), L-Methionine and L-Tryptophan (both at 40 μg/ml) was used for preparation of seed cultures and for both batch and continuous fermentations. The trace element stock solution consisted of 1.38 g ZnSO_4_·7H_2_O, 5.4 g FeCl_3_·6H_2_O, 1.80 g MnSO_4_·H_2_O, 0.17 g CuCl_2_, 0.56 g CoSO_4_·7H_2_O, 0.06 g H_3_BO_3 _and 10 ml 37% HCl per liter.

Both LB and M9 media were supplemented (when required) with ampicillin to a final concentration of 100 μg/ml.

### Cell cultivation

Competent *E. coli *LE392 cells were prepared by the calcium chloride procedure [28] and transformed with the above described expression plasmids.

#### Batch cultivation in flasks

*E. coli *LE392 cells transformed with the plasmids pΔP_1_-(ΔSD)-hIFNγ and pP_1_-(ΔSD)-hIFNγ were grown in M9 medium containing 100 μg/ml ampicillin in 100-ml Erlenmeyer flasks (working volume of 10 ml) at 37°C and 200 rpm to A_600 _= 0.7.

#### Cultivation in a bioreactor

*E. coli *LE392 cells transformed with the plasmids pP_1_-(SD)-hIFNγ, pP_1_-(4SD)-hIFNγ, pΔP_1_-(ΔSD)-hIFNγ and pP_1_-(ΔSD)-hIFNγ were used for batch and chemostat cultivations.

##### Inoculum

Single colonies of transformed *E. coli *LE392 cells were transferred to 100 ml flasks with 10 ml sterile M9 medium (pH 7.0) containing 100 μg/ml ampicillin. The flasks were incubated at 37°C and 200 rpm until an optical density of A_600 _= 1.5-1.8.

##### Fermentation conditions

Batch and chemostat cultivations were performed in M9 medium containing 1.96 g/L glucose (without antibiotic) in a Biostat^® ^Bplus Bioreactor (Sartorius BBI Systems) with a working volume of 600 ml. The pH value was maintained during cultivation at 7.0 ± 0.1 by 2M NaOH, temperature and stirrer speed were kept constant at 37°C and 600 rpm, respectively. Dissolved oxygen was monitored using a pO_2_-Elektrode Oyferm FDA160 (Hamilton), and pO_2 _was controlled at 80-90% of air saturation by the airflow. Feeding of chemostat cultivations was initiated after the initial batch phase (cell density of A_600 _= 1.5-1.6) at a constant dilution rate of 0.3 h^-1^. The inoculum in all experiments was 1% v/v of the final culture volume. Samples were aseptically collected at different time intervals and used for determination of cell concentration, plasmid copy number, glucose and hIFNγ quantification, as well as for plasmid stability assay.

### Analytical methods

#### Determination of cell concentration

Cell growth was monitored by measuring the optical density at 600 nm (in triplicates) using a Ultrospec 500 *pro *Visible Spectrophotometer (GE Healthcare Life Sciences). Optical density was converted to dry cell mass concentration using a standard curve determined before. To determine dry cell weight bacterial cells were collected from 5 ml cell suspension by centrifugation at 5000 × g for 5 min at 4°C, washed twice with distilled water and dried at 100°C to constant weight.

#### Plasmid stability assay

To determine the fraction of plasmid-harbouring cells, culture samples were appropriately diluted with 0.9% w/v NaCl, spread on LB-agar plates and incubated at 37°C for 12 h. Single colonies (250) were picked with sterile applicator sticks and transferred to LB-agar plates containing 100 μg/ml ampicillin. After 12 h at 37°C the colonies were counted and the segregational instability was represented as the ratio of colonies growing on ampicillin plates to the total number of transferred colonies (250).

#### Glucose and hIFNγ quantification

Samples of 1-5 ml bacterial culture were centrifuged at 5000 × g for 5 min at 4°C. The glucose concentration in the supernatant (in triplicates) was determined using a BioProfile 100 Plus Analyzer (Nova Biomedical). The harvested bacteria were lysed by boiling (5 min) in 1 ml 7M guanidine hydrochloride (GnHCl) and after appropriate dilution of the samples (so that they remained in the linear range of reading) the content of hIFNγ was determined by ELISA (in 6 repetitions for each probe) using the Ready-Set-Go! kit for human interferon gamma (NatuTec), following the manufacturer's instructions.

#### hIFNγ-mRNA determination

The relative content of hIFNγ-mRNA was determined by hybridization using a 19-nt ^32^P-labeled oligonucleotide specific for the hIFNγ gene as already described [33].

#### Determination of plasmid copy-number

Plasmid copy-number (**N**_**p**_) was determined by Real-time quantitative PCR (QPCR) as described by Lee et al. [32] using the chemostat cultures of *E. coli *LE392 transformed with the plasmids pP_1_-(SD)-hIFNγ, pP_1_-(4SD)-hIFNγ, pP_1_-(ΔSD)-hIFNγ and ΔP_1_-(ΔSD)-hIFNγ. Samples were derived 20 h after switch to continuous cultivation. Total DNA was isolated using a QIAamp^® ^DNA Mini Kit (Qiagen), following the method for bacterial cultures. Since all plasmids in the current study bear *bla *gene (target gene) and the host *E. coli *LE392 cells harbour chromosomal D-1-deoxyxylulose-5-phosphate synthase gene (*dxs*) (housekeeping gene) the same primers sets, calibrator (plasmid pGEM-BD, carrying both *bla *and *dxs *gene) and thermal cycling protocol as proposed by Lee et al. [32], were used. Real-time QPCR amplification was carried out in a Rotor-Gene™ instrument (Corbett Research, Qiagen) using MESA GREEN qPCR MasterMix Plus for SYBR^® ^Assay No ROX kit (Eurogentec) in 9 repetitions for each probe.

## Results and Discussion

To study the influence of transcription and translation on plasmid segregation four expression plasmids based on the cloning vector pBR322 were constructed. Plasmids were designed to express a synthetic hIFNγ under the control of different regulatory elements (see Methods) in order to vary the efficiency of transcription and translation. The plasmid pP_1_-(SD)-hIFNγ bears a strong constitutive promoter (P_1_) and a strong synthetic ribosome binding site (SD) thus insuring a high level of constitutive gene expression. In the construct pP_1_-(4SD)-hIFNγ a cluster of four identical SD sequences is substituted for the single SD in the previous plasmid. As shown earlier [34], the repetition of the SD sequence can have a strong suppressive effect on translation and therefore on the yield of recombinant protein. The constructs pΔP_1_-(ΔSD)-hIFNγ and pP_1_-(ΔSD)-hIFNγ are derivatives of the plasmid pP_1_-(SD)-hIFNγ in which both promoter and SD sequence (in pΔP_1_-(ΔSD)-hIFNγ) or the SD sequence (in pP_1_-(ΔSD)-hIFNγ) were deleted. Therefore, the hIFNγ gene in the first plasmid can not be transcribed and in the second plasmid can not be translated. To prove this experimentally *E. coli *LE392 cells transformed with the plasmids pΔP_1_-(ΔSD)-hIFNγ and pP_1_-(ΔSD)-hIFNγ were cultivated in flasks with M9 medium to A_600 _= 0.7 and the content of hIFNγ and hIFNγ-mRNA was measured as already described [33]. As expected, no hIFNγ-mRNA and no protein (hIFNγ) were detected in cells harbouring pΔP_1_-(ΔSD)-hIFNγ and pP_1_-(ΔSD)-hIFNγ, respectively.

**Figure 2:**
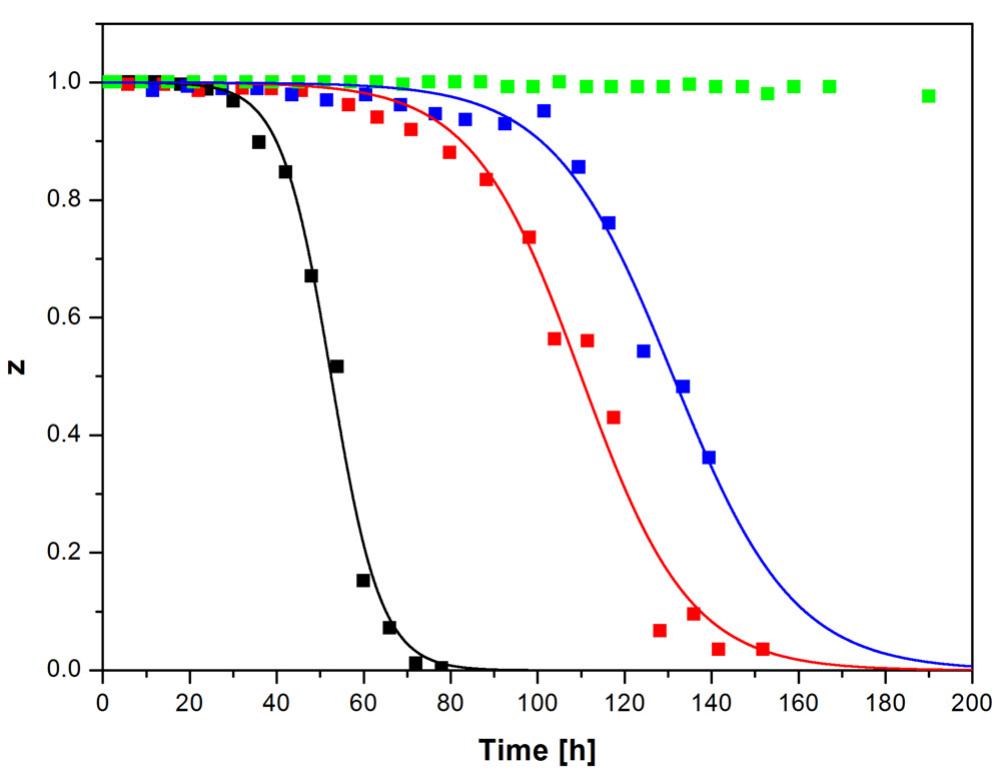
**Plasmid-harbouring cell fraction z as a function of cultivation time**. Experimental results were processed and simulations were carried out employing Eq. 23 and using the software product Berkeley Madonna, Version 8.3.9. (black squares and line - pP_1_-(SD)-hIFNγ; red squares and line - pP_1_-(4SD)-hIFNγ; blue squares and line - pP_1_-(ΔSD)-hIFNγ; green squares - pΔP_1_-(ΔSD)-hIFNγ. Data points and lines represent experimental results and trajectories predicted by the model calculations, respectively.)

**Figure 3:**
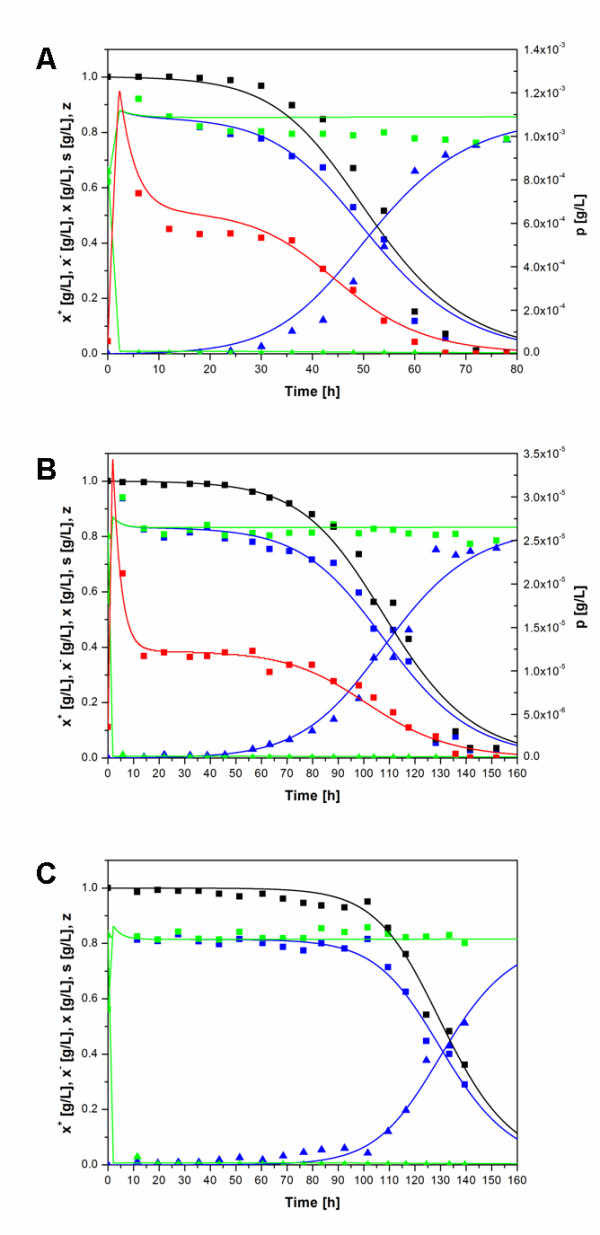
**Growth and plasmid loss kinetics of transformed *E. coli *LE392 cells in a chemostat culture**. Experimental data (data points) and simulation curves obtained by the model of Lee et al. (lines): plasmid-harbouring biomass, **x**^+ ^(blue squares and line); plasmid-free biomass, **x**^- ^(blue triangles and line); total biomass, **x **(green squares and line); glucose concentration, **s **(green triangles and line); hIFNγ concentration, **p **(red squares and line) and plasmid-harbouring cell fraction, **z **(black squares and line) for cells carrying the plasmid pP_1_-(SD)-hIFNγ (Figure **3A**), pP_1_-(4SD)-hIFNγ (Figure **3B**) and pP_1_-(ΔSD)-hIFNγ (Figure **3C**).

Plasmid loss kinetics was studied for the four constructs pP_1_-(SD)-hIFNγ, pP_1_-(4SD)-hIFNγ, pΔP_1_-(ΔSD)-hIFNγ and pP_1_-(ΔSD)-hIFNγ in M9 medium supplemented with glucose in chemostat cultivations. Following the initial batch phase, feeding was initiated at a cell density of A_600 _= 1.5-1.6 at a constant dilution rate of 0.3 h^-1^. The fraction of plasmid-harbouring cells in the total cell population versus cultivation time during the continuous phase for the construct pΔP_1_-(ΔSD)-hIFNγ is presented in Figure 2. Time dependence of this and other variables (plasmid-harbouring, plasmid-free and total biomass concentrations, concentration of hIFNγ in the culture volume, glucose concentration) during the continuous phase of cultivation of *E.coli *LE392 cells transformed with the plasmids pP_1_-(SD)-hIFNγ, pP_1_-(4SD)-hIFNγ and pP_1_-(ΔSD)-hIFNγ are shown in Figures 3A - C.

### Description of plasmid segregation by the model of Stewart & Levin

To describe the population dynamics of plasmid-harbouring and plasmid-free cells the nonlinear fitting method of Davidson et al. [35], which is based on the equations proposed by Stewart & Levin [30], was used (see Additional file 1: Appendix). These equations describe the growth kinetics of plasmid-harbouring and plasmid-free cells in chemostat culture (see Eq. 20 and Eq. 21), which can be combined into a single equation representing the fraction of plasmid-harbouring cells in the population **z **as a time dependent function (Eq. 23). Eq. 23 includes the parameters **Δ **(difference in the specific growth rate between plasmid-free and plasmid-harbouring cells as in Eq. 26) and **Θ **(specific rate of generation of plasmid-free cells, or specific plasmid loss rate as in Eq. 22). Employing Eq. 23 the values of **Δ **and **Θ **were estimated for the plasmids pP_1_-(SD)-hIFNγ, pP_1_-(4SD)-hIFNγ and pP_1_-(ΔSD)-hIFNγ (Table 2). As the fraction of plasmid-harbouring cells **z **did not change significantly during cultivation both parameters were not determined for the plasmid pΔP_1_-(ΔSD)-hIFNγ. The fraction of plasmid-harbouring cells in the total population **z **versus cultivation time is shown in Figure 2 for all investigated plasmids.

**Table 2:** Estimated values for Δ and Θ.

Parameter [Units]	Plasmid		
	
	**pP**_**1**_**-(SD)-hIFNγ**	**pP**_**1**_**-(4SD)-hIFNγ**	**pP**_**1**_**-(ΔSD)-hIFNγ**
Δ [h^-1^]	0.1741	0.0799	0.0723

Θ [h^-1^]	1.8632 × 10^-5^	1.2152 × 10^-5^	5.4836 × 10^-6^

### Kinetic description of cell growth and product formation by the model of Lee et al

The genetically structured model of Lee et al. [31] (see Additional file 1: Appendix) was employed to describe bacterial growth in a chemostat, hIFNγ production and plasmid loss kinetics of *E. coli *LE392 cells transformed with the plasmids pP_1_-(SD)-hIFNγ, pP_1_-(4SD)-hIFNγ and pP_1_-(ΔSD)-hIFNγ. Since recombinant hIFNγ is a non-secretion protein, the concept for intracellular product formation/inhibition, as given by Eq. 5, was used.

The model of Lee et al. involves numerous parameters whose values were: i) obtained by experiments; ii) taken after consideration; iii) calculated by fitting model equations to experimental data.

#### Parameter values determined by experiments

The maximum specific growth rate **μ**_**max **_in Eq. 4 and 5 refers to the host cells (plasmid-free). In the current study a parameter value of 0.66 h^-1 ^was estimated from the growth kinetics of *E. coli *LE392 cells cultivated in minimal (M9) medium supplemented with glucose in a batch reactor (data not shown).

The intracellular plasmid concentration **G**_**in **_(Eq. 5, Eq. 14) is a linear function of the average plasmid copy-number **N**_**p**_. For a representative plasmid size and *E. coli *cell volume **G**_**in **_can be calculated by Eq. 6 [31].

The model of Lee et al. is based on the assumption that the plasmid copy-number per cell remains constant during the cultivation. Employing QPCR [32] the copy-number of the investigated plasmids was measured during chemostat cultivations in M9 medium 20 h after initiation of feeding, i.e. when a quasi steady-state for the plasmid copy-number was established. The plasmid copy-number values determined under this condition (where the bacterial population consists of plasmid-harbouring cells only) is considered as corresponding to the **N**_**p **_in the model of Lee et al. The plasmid copy-number values corresponding to the constructs pP_1_-(SD)-hIFNγ, pP_1_-(4SD)-hIFNγ, pΔP_1_-(ΔSD)-hIFNγ and pP_1_-(ΔSD)-hIFNγ were 41, 24, 22 and 30, respectively.

The dilution rate **D **(Eq. 16-19), as well as the inlet substrate concentration **s**_**F **_(Eq. 18) were determined experimentally and remained constant for all chemostat cultivations.

#### Parameter values taken after consideration

The exponent **m **and the maximum intracellular plasmid concentration **G**_**inmax **_in Eq. 5 describe mathematically the inhibitory effect of the plasmid copy-number on cell growth. Both parameters might be highly dependent on some factors related to plasmid structure and host cells genotype, as well as on the cultivation conditions [31]. For *E. coli *Lee et al. considered 1 and 0.036 g/L as reasonable for **m **and **G**_**inmax **_respectively. This **G**_**inmax **_value corresponds to a plasmid copy-number of about 300 (Eq. 6). Palaiomyletou et al. [36] and Altinash et al. [37] have used similar values for **G**_**inmax**_, but different values for **m**, applying the model of Lee et al. to describe the cell growth and production of recombinant proteins in *E. coli *and *S. cerevisiae*. Palaiomyletou et al. set in their calculations m = 1, whereas Altinash et al. obtained better results with m = 0.01. The latter indicates that the **m **value is of great importance for the adequate cell growth modelling. In the following a **G**_**inmax **_of 0.036 g/L (as given by Lee et al.) was assumed. Using nonlinear fitting of the model equations to the experimental data obtained with the plasmid pP_1_-(ΔSD)-hIFNγ (see below), a value of 2.399 for **m **was determined. Since the plasmids used in this study have similar molecular characteristics (molecular mass and genetic structure), the same **m **value was also accepted for the plasmids pP_1_-(SD)-hIFNγ and pP_1_-(4SD)-hIFNγ.

Analogously, the exponent **n **and the maximal intracellular concentration of the recombinant product **p**_**inmax **_define the inhibitory effect of the recombinant product on the growth of plasmid-harbouring bacteria (Eq. 5). These two parameters can also be affected by other factors such as properties of the recombinant protein or the genotype of host bacteria [31]. Lee et al. proposed two values for the product inhibition exponent **n**: n = 0 (no product inhibition) and n = 1 (product inhibition). Based on the maximal recombinant product levels reported in literature, they have proposed 150 g/L for the parameter **p**_**inmax**_.

Yield of hIFNγ expressed in *E. coli *LE392 can approach 30% of the total cell protein [own data, not published]. Considering the gross chemical composition of *E. coli *cells (70% water, 15% proteins and 15% other compounds [38]), and assuming a cell density of 1000 g dry biomass/L dry biomass [31], the recombinant product concentration was estimated to be of about 150 g hIFNγ/L dry biomass, i.e. the same **p**_**inmax **_value as used by Lee et al. This parameter **p**_**inmax **_represents the maximal intracellular concentration of recombinant product at which the cell growth is impossible, i.e. μ^+ ^= 0 (Eq. 5). Assuming that the maximal intracellular product concentration experimentally determined corresponds to the theoretical value of **p**_**inmax**_, 150 g/L and 1 were assumed for **p**_**inmax **_and **n**, respectively.

The Monod constant **K**_**s **_in Eq. 4 and 5 is assumed to be equal for both plasmid-harbouring and plasmid-free cells, as proposed in [31]. In the following, **K**_**s **_was set as 0.005 g/L, as proposed by Atkinson et al. [39] for *E. coli *grown in minimal medium containing glucose.

For the overall transcription and translation rate parameters **k_p_^0 ^**and **k_q_^0 ^**(both growth rate-dependent), as well as for the mRNA decay constant **k**_**d **_(Eq. 11), the values obtained by Lee et al. for the wild-type λdv plasmid replicon [40,41] were used. Furthermore, it was assumed that the cell density parameter **ρ**_**B **_(Eq. 13) had the same value as in [31], and that the recombinant protein degradation rate constant **k**_**e **_(Eq. 10) is negligible. All parameter values are summarized in Table 3.

**Table 3:** Summary of parameter values used for model simulations.*

Parameter	Value	Units
D	0.3	h^-1^

G_inmax_	0.036	g plasmid DNA/L biomass

K_s_	0.005	g/L

k_d_	0.46	min^-1^

k_e_	0	min^-1^

k_p_^0^	2400/(233 μ^-2 ^+ 78)	min^-1^**

k_q_^0^	3600α/(82.5 μ^-1 ^+ 145)	min^-1^**
	α = 1 for μ > ln2	
	α = μ/ln2 for μ < ln2***	

m	2.399****	-

N_p_	41 (pP_1_-(SD)-hIFNγ)	-
	24 (pP_1_-(4SD)-hIFNγ)	
	30 (pP_1_-(ΔSD)-hIFNγ)	

n	1	-

p_inmax_	150	g protein/L dry biomass

s_F_	1.96	g/L

μ_max_	0.66	h^-1^

ρ_B_	1000	g dry biomass/L dry biomass

*In the absence of translation (as in pP_1_-(ΔSD)-hIFNγ) the gene expression parameters are ignored.

**Calculating **k_p_^0 ^**and **k_q_^0 ^**the specific growth rate **μ **is expressed in h^-1^.

***By α = μ/ln2 (for μ < ln2) and Eq. (9), the substitution of **k_p_^0^**, **k_q_^0 ^**and **k**_**d **_in Eq. 11 results in Eq. 15.

****The **m **value was determined by nonlinear fitting for pP_1_-(ΔSD)-hIFNγ and set in the calculations for both pP_1_-(SD)-hIFNγ and pP_1_-(4SD)-hIFNγ (see below).

#### Parameters determined by fitting model equations to experimental data

To determine the exponent **m **describing the plasmid vector inhibition experimental data for pP_1_-(ΔSD)-hIFNγ were used. Since the hIFNγ gene in this construct is transcribed but the hIFNγ-mRNA is not translated, the Monod equation describing the specific growth rate of plasmid-harbouring cells (Eq. 5) is simplified and the kinetic relation for recombinant product formation (Eq. 14) drops off.

The yield factor **Y**_**x/s **_(Eq. 3) is assumed to be the same for both plasmid-harbouring and plasmid-free cells [31]. Since substrate consumption necessary for formation of the recombinant product is low it can be neglected. For the sake of simplicity substrate consumption for the endogenous cell metabolism was also neglected. By batch cultivations of both non-transformed and plasmid-bearing *E.coli *LE392 cells a yield factor Y_x/s _= 0.5 g biomass/g glucose was obtained. Different **Y**_**x/s **_values were expected however, for chemostat cultivations depending on the dilution rate. Seo and Bailey have shown experimentally that this parameter decreases with increasing dilution rate, which contradicts the theoretical expectations [42].

The parameter **γ **in Eq. 12 represents the efficiency of transcription and translation of the cloned gene and therefore it should have different values for the plasmids pP_1_-(SD)-hIFNγ and pP_1_-(4SD)-hIFNγ. However, for pΔP_1_-(ΔSD)-hIFNγ (devoid of promoter) and pP_1_-(ΔSD)-hIFNγ (lacking SD sequence) γ = 0 because of the lack of transcription and translation, respectively.

The relative plasmid segregation rate **θ**, which is a key parameter in analyzing plasmid segregation, is assumed to be constant in the model of Lee et al. [31].

The values of **θ**, **Y**_**x/s **_and **γ **for the constructs pP_1_-(SD)-hIFNγ, pP_1_-(4SD)-hIFNγ and pP_1_-(ΔSD)-hIFNγ, and the exponent **m **for the plasmid pP_1_-(ΔSD)-hIFNγ were determined numerically using experimental data obtained from continuous cultivations. To this aim the model equation system was fitted to the data for the plasmid-harbouring biomass **x**^+^, overall recombinant product concentration **p **and plasmid-harbouring cell fraction **z **(= x^+^/x) using the software product Berkeley Madonna, Version 8.3.9. The initial conditions (experimental data determined immediately before starting continuous cultivations) are listed in Table 4.

**Table 4:** Initial values used for parameter estimation.

Variable [Units]	Plasmid construct		
	
	**pP**_**1**_**-(SD)-hIFNγ**	**pP**_**1**_**-(4SD)-hIFNγ**	**pP**_**1**_**-(ΔSD)-hIFNγ**
x^+^(0) [g/L]	0.62	0.56	0.56

x^-^(0) [g/L]	0	0	0

s [g/L]	0.66	0.8	0.83

p [g/L]	5.78 × 10^-5^	3.54 × 10^-6^	-

Experimental data and model simulations for plasmid-harbouring, plasmid-free and total biomass, limiting substrate, recombinant product and plasmid-harbouring cell fraction for the plasmids pP_1_-(SD)-hIFNγ, pP_1_-(4SD)-hIFNγ and pP_1_-(ΔSD)-hIFNγ are shown in Figures 3A-C, respectively.

The calculated curves obtained by the model of Lee et al. [31] fit well the experimental data for all investigated plasmids. The root mean square deviation (RMSV) was 0.137, 0.116 and 0.057 for the plasmids pP_1_-(SD)-hIFNγ, pP_1_-(4SD)-hIFNγ and pP_1_-(ΔSD)-hIFNγ, respectively.

The estimated values of the parameters **θ**, **Y**_**x/s **_and **γ **are listed in Table 5. A value of 2.399 for **m **was determined for the plasmid pP_1_-(ΔSD)-hIFNγ.

**Table 5:** Parameter values estimated numerically by the model of Lee et al.

Parameter	Plasmid construct		
	
	**pP**_**1**_**-(SD)-hIFNγ**	**pP**_**1**_**-(4SD)-hIFNγ**	**pP**_**1**_**-(ΔSD)-hIFNγ**
θ	7.435 × 10^-4^	1.829 × 10^-4^	4.488 × 10^-6^

Y_x/s_	0.438	0.426	0.417

γ	0.110	0.0036	-

The gene expression parameter **γ **(reflecting transcription and translation efficiency, see Eq. 12) depends on the strength of both promoter and ribosome binding site [31]. In particular the replacement of the single SD sequence in pP_1_-(SD)-hIFNγ with a tetrameric SD sequence (as in the construct pP_1_-(4SD)-hIFNγ) led to a sharp decrease in the yield of hIFNγ (Figures 3A and 3B). As seen from Table 5, the predicted γ values for the plasmids pP_1_-(SD)-hIFNγ and pP_1_-(4SD)-hIFNγ correlate well with the experimentally observed reduction in the yield of hIFNγ.

The model predicts almost identical values of the yield factor **Y**_**x/s **_at continuous cultivation conditions for all investigated plasmids (about 0.43 g biomass/g glucose, Table 5).

The plasmid loss probability **θ **predicted by the model of Lee et al. (Table 5) clearly demonstrates that the alterations in the ribosome binding site (affecting the efficiency of hIFNγ-mRNA translation) interfere with the segregational plasmid stability. A reverse correlation between the yield of recombinant protein and the segregational plasmid stability was observed.

### Plasmid segregation

The plasmids pΔP_1_-(ΔSD)-hIFNγ, pP_1_-(SD)-hIFNγ, pP_1_-(4SD)-hIFNγ and pP_1_-(ΔSD)-hIFNγ carrying different gene expression control elements were studied to evaluate the role of heterologous gene expression on plasmid segregation. Results demonstrated that altered control elements led to well distinguished differences in the population dynamics between plasmid-harbouring and plasmid-free cells (Figure 2).

Mathematically, the cell population dynamics is influenced mainly by the following two factors: a) Difference in the specific growth rate between plasmid-harbouring and plasmid-free cells **Δ **and b) Probability of generation of plasmid-free cells **Θ **due to the plasmid loss. The latter itself is a function of the specific growth rate of the plasmid-harbouring cells **μ**^+ ^and the relative plasmid loss rate **θ **(Eq. 22). In some models [30,43,44]** Δ **and **Θ **are expressed as constants assuming apparent steady-state conditions. In general, however, these parameters are complex functions depending on cell genetics and cell physiology, as well as on the corresponding cultivation conditions [45]. In the model of Lee et al. **Δ **and **Θ **are considered to be *functions*. The specific growth rate of plasmid-free cells is described by the Monod-equation (Eq. 4), whereas the corresponding rate of plasmid-harbouring cells depends on the cellular content of both recombinant protein and expression plasmid (Eq. 5). In the model of Lee et al. the dimensionless probability of plasmid loss **θ **describing plasmid segregation is assumed to be a *constant *irrespective of the specific growth rate of host cells and cultivation conditions [31]. In the experimental system established here the four plasmids carry the same reporter gene (hIFNγ) but different genetic elements (promoter and SD sequence) regulating its expression. The altered expression levels (transcription and/or translation) of the recombinant gene led to variations in both the yield of hIFNγ and average plasmid copy-number per cell. Therefore, it is assumed that these two factors are responsible for the observed differences in the population dynamics between plasmid-harbouring and plasmid-free cells.

#### Population dynamics of *E. coli* LE392 cells transformed with the plasmids pP_1_-(SD)-hIFNγ, pP_1_-(4SD)-hIFNγ and pP_1_-(ΔSD)-hIFNγ

The population dynamics of cells transformed with pP_1_-(SD)-hIFNγ, pP_1_-(4SD)-hIFNγ and pP_1_-(ΔSD)-hIFNγ was analyzed by the models of Stewart & Levin and of Lee et al.

##### Difference in the specific growth rate between plasmid-free and plasmid-harbouring cells (**Δ**)

**Figure 4:**
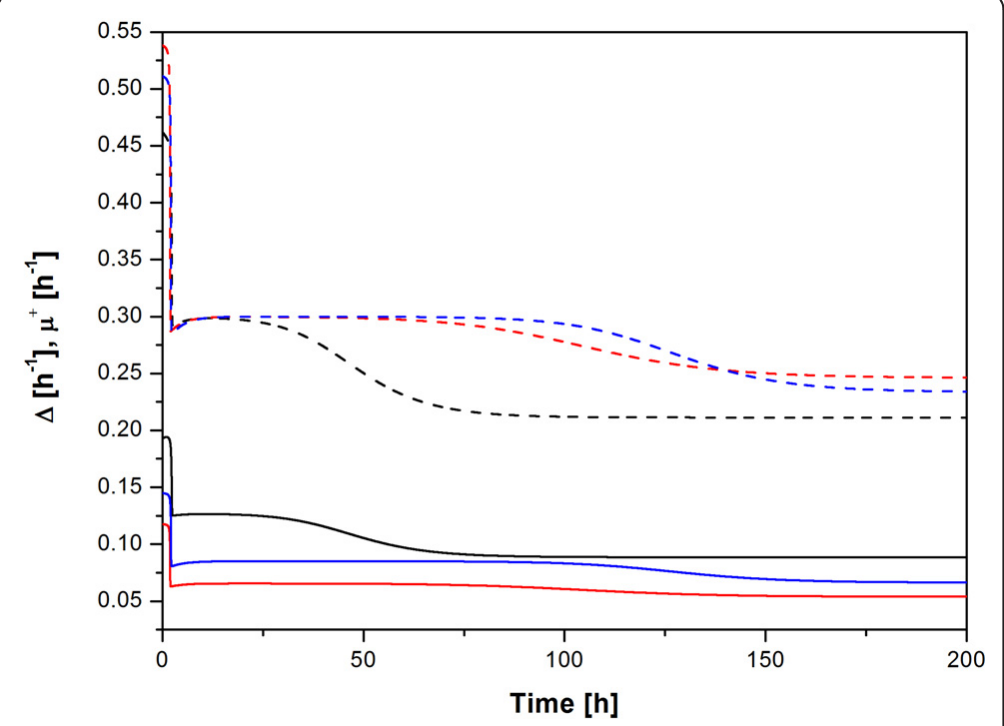
**Simulated specific growth rate difference and specific growth rate of plasmid-harbouring cells**. Specific growth rate difference **Δ **(solid lines) and specific growth rate of plasmid-harbouring cells **μ**^+ ^(dashed lines) as a function of cultivation time predicted by the model of Lee et al. for the plasmids pP_1_-(SD)-hIFNγ (black lines), pP_1_-(4SD)-hIFNγ (red lines) and pP_1_-(ΔSD)-hIFNγ (blue lines).

The values of **Δ **were calculated by the equations of Stewart & Levin (Table 2). As seen from this table, the highest **Δ **value was observed for the plasmid pP_1_-(SD)-hIFNγ and this is correlated with the highest cellular content of recombinant product (hIFNγ) and the highest plasmid copy-number compared to the other two plasmids. Simulations performed using the model of Lee et al. also confirmed the highest specific growth rate difference between cells harbouring pP_1_-(SD)-hIFNγ and plasmid-free cells (Figure 4).

These results agree with the fact that **Δ **increases with increasing the yield of recombinant protein and plasmid content in the cell, which is related to the increased metabolic burden of the plasmid-harbouring cells [22-25].

##### Relative (**θ**) and specific (**Θ**) plasmid loss rates

**Figure 5:**
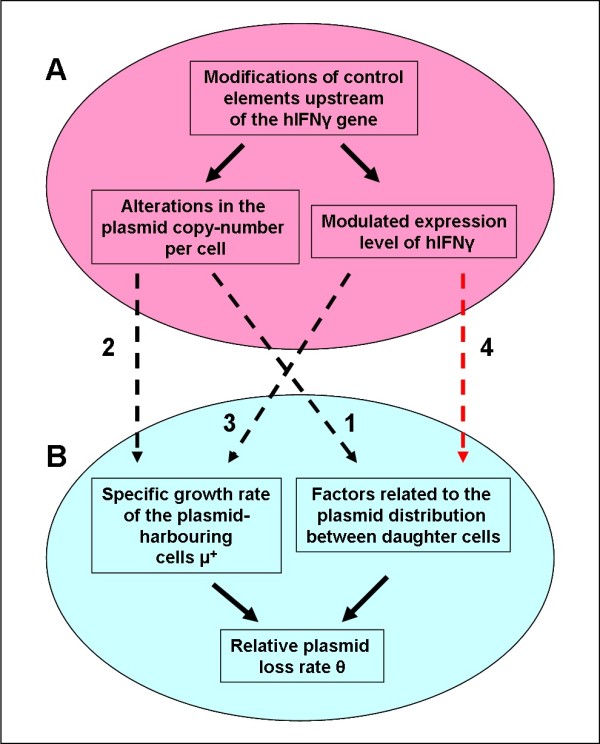
**Relationship between recombinant gene control elements and relative plasmid loss rate**. Interconnections between the effects caused by the modifications of the recombinant gene control elements (**A**) and factors influencing the relative plasmid loss rate **θ **(**B**).

The probability of plasmid loss **θ **was determined by a nonlinear fitting procedure employing the model of Lee et al., (Table 5). The obtained results clearly demonstrate that the activity of the genetic elements (promoter and SD sequence) regulating hIFNγ gene expression determines (directly or indirectly) the plasmid loss probability **θ**. As already mentioned, the genetic modification of these elements resulted in different gene expression levels (yield of recombinant protein) and different plasmid copy-number (Figure 5A). The question rises of how these two factors affect plasmid loss probability **θ**.

In general, **θ **is affected by various factors related with plasmid distribution between the daughter cells during cell division or to the specific growth rate of the plasmid-harbouring cells (Figure 5B).

Among the factors affecting plasmid partitioning (Figure 5B) are the plasmid copy-number **N**_**p **_[9], plasmid multimerization [4], concatameric replication [13], presence of partitioning elements in the plasmid [4], host cell genotype [21], etc. In the focus of this study was the average (related to the whole cell population) plasmid copy-number (Figure 5A), whose reduction often leads to segregational plasmid instability, i.e. to an increased plasmid loss (**θ**) (see Figure 5, *Arrow 1*), [46]. Based on the presented results, however, it seems unlikely that the variations in the segregational instability of the investigated plasmids are related to differences in the copy-number. In especially, there seems to be no correlation between plasmid content and the relative plasmid loss rate **θ**. Moreover, the highest plasmid copy-number value was determined for the plasmid pP_1_-(SD)-hIFNγ (Table 3), showing the highest **θ **value (Table 5).

It is clear that the level of gene expression and plasmid copy-number interfere with the specific growth rate of plasmid-harbouring cells. Both high plasmid copy-number and high yield of recombinant protein result in a decrease in specific growth rate of the plasmid-harbouring cells **μ**^+ ^(Figure 5, *Arrows 2 and 3*) as described in the model of Lee et al. by Eq. 5. As a result the decreased specific growth rate **μ**^+ ^leads to a decrease in the relative plasmid loss rate **θ**, respectively to increased plasmid stability [47]. However, the observed variation in stability/instability of the investigated plasmids cannot be explained satisfactorily by the difference in the specific growth rate of plasmid-harbouring cells in relation with the level of gene expression and plasmid copy-number. The highest **Δ **value determined by the model of Stewart & Levin (respectively the lowest value of **μ**^+^, according to Eq. 26) for the plasmid pP_1_-(SD)-hIFNγ should result in a minimal **θ **value. Conversely, a high plasmid loss rate should be expected for the plasmids pP_1_-(4SD)-hIFNγ and pP_1_-(ΔSD)-hIFNγ (both characterized by high **μ**^+ ^values). In contrast, however, no correlation between **μ**^+ ^and plasmid loss probability **θ **(as proposed by Mosrati et al. [47]) was observed.

**Figure 6:**
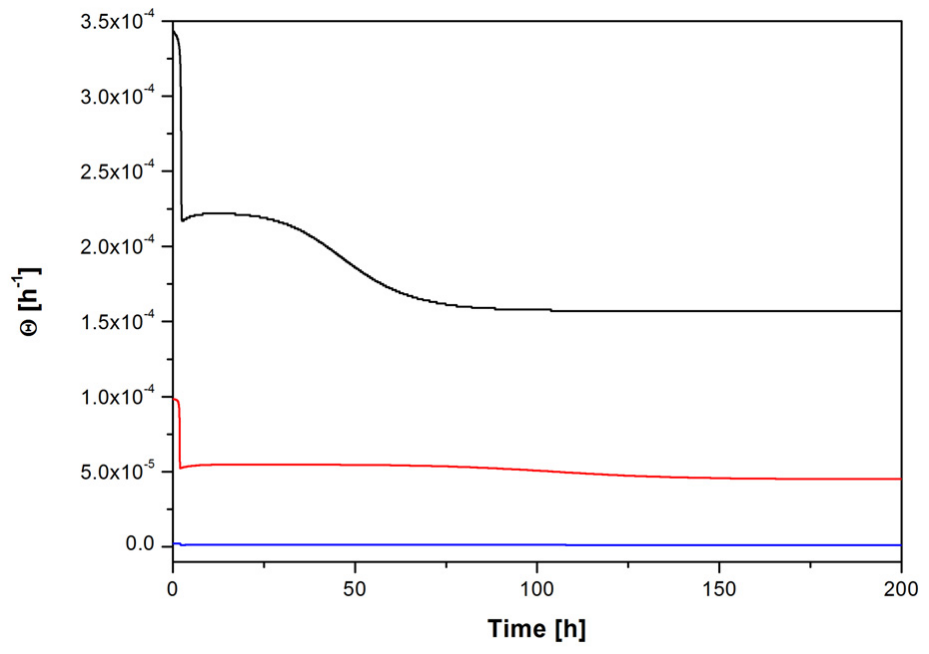
**Specific rate of generation of plasmid-free cells Θ simulated by the model of Lee et al**. The simulated graphs of **Θ **for pP_1_-(SD)-hIFNγ, pP_1_-(4SD)-hIFNγ and pP_1_-(ΔSD)-hIFNγ are presented with black, red and blue line, respectively.

**μ**^+ ^as well as the specific rate of generation of plasmid-free cells **Θ **can also be presented as time-dependent functions applying the model of Lee et al. **μ**^+ ^predicted by the model of Lee et al. (which corresponds to the results obtained by the model of Stewart & Levin) clearly shows that the specific growth rates of the cells transformed with the plasmids pP_1_-(4SD)-hIFNγ and pP_1_-(ΔSD)-hIFNγ are almost equal and higher than those of the cells transformed with the construct pP_1_-(SD)-hIFNγ (Figure 4). The time course of **Θ **according to the model of Lee et al. (Figure 6) confirms the tendency already reported for **Θ **values obtained by the model of Stewart and Levin (Table 2).

The observed relative plasmid loss rate (**θ**) of the expression plasmids used in this study is difficult to be explained by either the alterations in the plasmid copy-number (Figure 5, *Arrows 1 *and *2*) or gene expression efficiency (affecting **μ**^+^; see Figure 5, *Arrow 3*). This is an indication for the existence of other factors that might interfere with plasmid segregation. It can be assumed that hIFNγ gene expression influences significantly plasmid segregation (i.e. the probability of plasmid loss **θ**) on the level of plasmid distribution during cell division (Figure 5, *Arrow 4*). The latter might be related with the specificity of prokaryotic gene expression itself. Unlike in eukaryotes, the three processes replication, transcription and translation in prokaryotes are conjugated and all occur in one compartment (bacterial cytoplasm). This enables interactions between molecules that are principally engaged in different processes (replication, transcription or translation). It is shown for instance that the initiation of the chromosomal DNA replication in *E. coli *is dependent on transcriptional activation [48] where a direct interaction between DnaA (a bacterial replication initiator protein) and RNA polymerase is found [49,50]. Moreover, Szambowska et al. [51] provide evidence that during the initiation of λ phage DNA replication the λO protein (a replication initiator of phage λ) interacts directly with the β subunit of the bacterial RNA polymerase. Therefore, it might be assumed that especially in the presence of a strong constitutive promoter and a strong SD sequence (i.e. in case of extensive gene expression) the plasmid is easily involved in a very complex aggregate consisting of replicating plasmid, growing mRNA(s), translating ribosomes/polysomes and growing polypeptide chains. Apparently, the risk of a non-random distribution of such huge complexes between the daughter cells is much greater compared to the naked (silent) plasmids and might be influenced by many other factors. Among the latter are the structure and aminoacid composition of the recombinant protein, its solubility in the bacterial cytoplasm, the affinity to the cell membrane, etc. If the growing polypeptide chain is hydrophobic or bears an N-terminal secretion signal, it might "stick" to the plasma membrane. Membrane association on the other hand is a predisposition for a non-random partitioning and changes in the probability for appearance of plasmid-free cells and therefore relative plasmid loss rate **θ**. A potential factor promoting non-random plasmid partitioning could also be the formation of inclusion bodies (huge intracellular aggregates of unfolded recombinant protein), typical for the expression of many eukaryotic genes in bacteria. Usually, they have polar localization and could entangle growing polypeptide chains.

#### Population dynamics of *E. coli* LE392 cells transformed with the plasmid pΔP_1_-(ΔSD)-hIFNγ

The plasmid pΔP_1_-(ΔSD)-hIFNγ lacks the promoter and therefore the inserted hIFNγ gene is not active. As seen in Figure 2, this plasmid demonstrated an extremely high segregational stability (the fraction of plasmid-free cells in the population after 190 h of cultivation did not exceed 5%). The same figure shows that the next stable plasmid is the construct pP_1_-(ΔSD)-hIFNγ (devoid of a SD sequence), where the recombinant protein is also not synthesized. Comparing the obtained results one can estimate the impact of transcription only. Figure 2 shows that the inactivation of translation (deletion of the SD sequence) shifts the segregation curve to the right. However, compared to the hIFNγ producing plasmids (pP_1_-(SD)-hIFNγ and pP_1_-(4SD)-hIFNγ), this does not prevent the plasmid segregation. The latter happens only after interrupting the transcription. This phenomenon might be explained by a mechanism proposed by Stueber & Bujard [26], who showed that extensive transcription interferes with plasmid replication and leads to a reduction in plasmid copy-number and segregational plasmid instability [27,28]. In this study, however, the plasmid copy-number of the transcriptionally inactive construct (pΔP_1_-(ΔSD)-hIFNγ) was lower (22 copies per cell) compared to that of the other three plasmids, i.e. the complete inactivation of the hIFNγ gene did not result in an increase in plasmid copy-number but had a strong stabilizing effect against segregation. Apparently this result cannot be explained by the above mentioned mechanism and agrees well with the hypothesis raised in this study about the possible role of the extensive constitutive gene expression on plasmid segregation. When interpreting these results, however, one should take into consideration that the data obtained by Stueber & Bujard [26] refer to chloramphenicolacetyltransferase (CAT) expressing plasmids and those presented above concern plasmids expressing a hIFNγ gene. In both cases a high level of expression can be achieved (up to 30-40% of the total protein) but the two proteins differ in their solubility in bacterial cytoplasm (CAT is soluble, whereas the hIFNγ forms inclusion bodies). In future studies (already undertaken) we aim to shed more light on the relationship between plasmid stability/instability and the intracellular state of the expressed protein.

### Results of simulation studies using the model of Lee et al

The model of Lee et al. was employed for simulations (see Additional file 2: Simulation studies) to study different trends that can be expected for chemostat cultivation of *E. coli *LE392 cells transformed with the plasmid pP_1_-(SD)-hIFNγ at different dilution rates.

## Conclusions

Transcription and translation of hIFNγ gene interfere with plasmid segregation. Switching-off transcription protects ColE1-like plasmids against segregation. However, the plasmid copy-number is not increased compared to other plasmid constructs tested. An increase in constitutive gene expression decreases the segregational plasmid stability (i.e. increases the relative plasmid loss rate). However, this is neither due to a decrease in the plasmid copy-number nor to an increase in the specific growth rate of the plasmid-harbouring cells. Therefore, constitutive gene expression seems to be a major factor interfering with ColE1-like plasmid segregation. A model of Lee et al. (1985), which describes growth and product formation kinetics of recombinant *E. coli *LE392 cells expressing constitutively hIFNγ in chemostat culture, allowed fitting of experimental data. To our knowledge, this is the first application of the model of Lee et al. for experimental studies of plasmid segregation in chemostat culture.

## Nomenclature

D - dilution rate      [h^-1^]

G_in _- intracellular plasmid concentration   [g plasmid/L biomass]

G_inmax _- maximal intracellular plasmid concentration [g plasmid/L biomass]

k_d _- decay constant of cloned gene messenger RNA  [min^-1^]

k_e _- decay constant of recombinant protein   [min^-1^]

k_p_^0 ^- overall transcription rate constant   [min^-1^]

k_q_^0 ^- overall translation rate constant    [min^-1^]

K_s _- Monod constant      [g/L]

m - exponent of plasmid vector inhibition

m_in _- intracellular concentration of cloned gene messenger RNA      [moles RNA/L biomass]

n - exponent of product inhibition term

N_p _- average number of plasmids per cell

p - concentration of recombinant protein related to the culture volume     [g protein/L culture broth]

p_in _- intracellular concentration of recombinant protein     [g protein/L dry biomass]

p_inmax _- maximal intracellular concentration of recombinant protein     [g protein/L dry biomass]

p_0 _- initial concentration of recombinant protein related to the culture volume     [g protein/L culture broth]

s - concentration of limiting nutrient (glucose)  [g/L]

s_F _- concentration of substrate (glucose) in the fresh nutrient medium     [g/L]

s_0 _- initial concentration of substrate (glucose)  [g/L]

t - time       [h]

x - total biomass concentration (=x^+ ^+ x^-^)   [g/L]

x^+ ^- concentration of plasmid-harbouring cells  [g/L]

x^- ^- concentration of plasmid-free cells   [g/L]

x_0_^+ ^- initial concentration of plasmid-harbouring cells [g/L]

x_0_^- ^- initial concentration of plasmid-free cells  [g/L]

Y_x/s _- biomass/substrate yield factor    [g biomass/g substrate]

z - plasmid-harbouring cell fraction (=x^+^/x)

### Greek symbols

Δ - difference in the specific growth rate between plasmid-free and plasmid-harbouring cells   [h^-1^]

γ - gene expression parameter

η - transcription efficiency     [moles RNA/g plasmid]

θ - relative plasmid loss rate

Θ - specific plasmid loss rate     [h^-1^]

μ^+ ^- specific growth rate of plasmid-harbouring cells  [h^-1^]

μ^- ^- specific growth rate of plasmid-free cells  [h^-1^]

μ* - specific growth rate, defined by Eq. 9   [h^-1^]

μ_max _- maximal specific growth rate of wild-type cells [h^-1^]

ξ - translation efficiency     [g protein/moles RNA]

ρ_B _- cell density     [g dry biomass/L dry biomass]

## Authors' contributions

MP carried out all kinetics studies and computer simulations and did the work related with bacterial cultivation in chemostat as well as most of the analytical procedures. He also summarized the results in the draft form of this manuscript. MP and SP performed the plasmid cloning. SP assisted in hIFNγ quantification and Real time PCR experiments. GN performed most of the immunochemical work and experiments related with the protein analysis. II designed the expression plasmids and coordinated the work on their construction. UR supervised the performance of the experimental work on bacterial cultivation, kinetic studies and contributed to the mathematical data analysis. MP, SP, GN, II and UR worked also on the preparation of this manuscript, which was finally approved and accepted by all authors.

## Supplementary Material

Additional file 1 **Appendix**. Mathematical model for description of bacterial growth and product formation kinetics, proposed by Lee et al. and mathematical model of Stewart & Levin. Click here for file

Additional file 2 **Results of simulation studies using the model of Lee et al.**. Simulations performed by the model of Lee et al. to study growth and hIFNγ formation kinetics of *E. coli *cells cultivated in a chemostat at different dilution rates. Click here for file
