# Supplementary material for: Effects of a recombinant gene expression on ColE1-like plasmid segregation in Escherichia coli
Source: BMC Biotechnol. 2011 Mar 1;11:18. doi: 10.1186/1472-6750-11-18 (PMC3061898; doi:10.1186/1472-6750-11-18)
Supplement: Additional file 1 — Appendix. Mathematical model for description of bacterial growth and product formation kinetics, proposed by Lee et al. and mathematical model of Stewart & Levin. [file 1472-6750-11-18-S1.PDF]

Appendix

Mathematical model for bacterial growth and product

Bacterial growth and product formation kinetics were described by the mathematical model of Lee et al. [31]. This model describes kinetic relations for plasmid-harbouring and plasmid-free biomass, limiting substrate and recombinant product, used in the relevant material balances.

Kinetic relations

The following kinetic relations for biomass and limiting substrate correspond to the case of pure segregational plasmid instability, i.e. the bacterial population is assumed to consist only of two cell types – plasmid-harbouring and plasmid-free cells:

Plasmid-harbouring biomass: $$r_{x^+} = \frac{dx^+}{dt} = \mu^+ \cdot x^+ - \theta \cdot \mu^+ \cdot x^+ \qquad (1)$$

where $\mathbf{x^+}$ is the concentration of plasmid-harbouring biomass, $\boldsymbol{\mu}^+$ is the specific growth rate of the plasmid-harbouring cells and $\boldsymbol{\theta}$ denotes the relative plasmid loss rate (i.e. a ratio of cloning vector loss rate to the specific growth rate of the plasmid-harbouring cells).

Plasmid-free biomass: $$r_{x^-} = \frac{dx^-}{dt} = \mu^- \cdot x^- + \theta \cdot \mu^+ \cdot x^+ \qquad (2)$$

$\mathbf{x^-}$ is the concentration of plasmid-free biomass and $\boldsymbol{\mu}^-$ is the specific growth rate of the plasmid-free cells.

Limiting substrate: $$r_s = \frac{ds}{dt} = -\frac{1}{Y_{x/s}} \cdot \left(r_{x^+} + r_{x^-} \right) \qquad (3)$$

s is the limiting substrate concentration in the broth. It is assumed that substrate consumption for synthesis of the recombinant product is negligible

and that the yield factor $\mathbf{Y_{x/s}}$ is identical for both plasmid-harbouring and plasmid-free cells.

Specific growth rate of plasmid-free and plasmid-harbouring cells:

The specific growth rate of the plasmid-free cells is described by the classical Monod equation:

$$\mu^{-} = \mu_{\max} \cdot \left(\frac{s}{K_s + s} \right) \qquad (4)$$

where $\boldsymbol{\mu_{max}}$ is the maximal specific growth rate of the host cells and $\mathbf{K_s}$ is the Monod constant.

Lee et al. have proposed an "extended" Monod equation, where the specific growth rate of the plasmid-harbouring cells is also a function of the intracellular concentration of recombinant protein $\mathbf{p_{in}}$ and the intracellular plasmid concentration $\mathbf{G_{in}}$:

$$\mu^{+} = \mu_{\max} \cdot \left(1 - \frac{G_{in}}{G_{in\max}} \right)^{m} \cdot \left(1 - \frac{p_{in}}{p_{in\max}} \right)^{n} \cdot \left(\frac{s}{K_s + s} \right) \qquad (5)$$

The terms $\left(1 - \frac{G_{in}}{G_{in\max}} \right)^{m}$ and $\left(1 - \frac{p_{in}}{p_{in\max}} \right)^{n}$ represent the inhibitory effect on cell growth of the plasmid content and recombinant protein, respectively. The parameters $\mathbf{G_{inmax}}$ and $\mathbf{p_{inmax}}$ denote maximal intracellular concentrations of plasmid DNA and recombinant protein at which the cell growth is not more possible, i.e. $\mu^{+} = 0$. **m** and **n** are exponents for plasmid and recombinant product inhibition. It is assumed that both plasmid-harbouring and plasmid-free cells have equal Monod constant $\mathbf{K_s}$. The plasmid concentration $\mathbf{G_{in}}$ [g/L] for representative plasmid size and cell volume of *E. coli* can be calculated using the following relationship [31]:

$$G_{in} = 0.0001186 \cdot N_p \qquad (6)$$

where $\mathbf{N_p}$ is the average plasmid copy-number per cell.

Recombinant product:

Assuming a first order kinetics for the decay of mRNA and the recombinant protein, Lee et al. have proposed the following material balances for the intracellular concentrations of both cloned gene mRNA ($\mathbf{m_{in}}$) and recombinant protein ($\mathbf{p_{in}}$):

$$\frac{dm_{in}}{dt} = k_p^0 \cdot \eta \cdot G_{in} - k_d \cdot m_{in} - \mu^* \cdot m_{in} \qquad (7)$$

$$\frac{dp_{in}}{dt} = k_q^0 \cdot \xi \cdot m_{in} - k_e \cdot p_{in} - \mu^* \cdot p_{in} \qquad (8)$$

$\boldsymbol{\mu}^*$ denotes overall specific growth rate of plasmid-harbouring biomass that describes growth of plasmid-harbouring cells by simultaneous appearance of plasmid-free cells, i.e.:

$$\mu^* = \mu^+ \cdot (1 - \theta) \qquad (9)$$

$\boldsymbol{\eta}$ and $\boldsymbol{\xi}$ denote transcription efficiency and translation efficiency, respectively. $\mathbf{k_p^0}$, $\mathbf{k_q^0}$, $\mathbf{k_d}$ and $\mathbf{k_e}$ are overall transcription rate constant, overall translation rate constant, decay constant of cloned-gene mRNA, and decay constant of recombinant protein, respectively. A quasi-steady-state approximation applied to $\mathbf{m_{in}}$ (i.e. $dm_{in}/dt = 0$) and a substitution of Eq. 7 in Eq. 8 leads to the following kinetic relation for intracellular recombinant product formation:

$$r_{p_{in}} = \frac{dp_{in}}{dt} = f\left(\mu^*\right) \cdot \gamma \cdot G_{in} - k_e \cdot p_{in} - \mu^* \cdot p_{in} \qquad (10)$$

where the function $\mathbf{f(\mu^*)}$ is defined by

$$f(\mu^*) = \frac{k_p^0 \cdot k_q^0}{k_d + \mu^*} \tag{11}$$

and the gene expression parameter $\boldsymbol{\gamma}$ by

$$\gamma = \eta \cdot \xi \tag{12}$$

The concentration of the recombinant product in the culture volume **p** can be calculated using the intracellular recombinant product concentration $\mathbf{p_{in}}$ by the following equation:

$$p = \frac{p_{in} \cdot x^+}{\rho_B} \tag{13}$$

where $\boldsymbol{\rho_B}$ is the cell density [cell mass/unit volume cell].

After differentiation of Eq. 13 by ρ_B=const and substitution of Eq. 10, the following kinetic relation for recombinant product concentration in the culture volume is obtained:

$$r_p = \frac{dp}{dt} = \frac{1}{\rho_B} \cdot \left[f\left(\mu^*\right) \cdot \gamma \cdot G_{in} \right] \cdot x^+ - k_e \cdot p \tag{14}$$

Using characteristic average values of the model parameters $\mathbf{k_p^0}$, $\mathbf{k_q^0}$ and $\mathbf{k_d}$ for recombinant gene expression in *E. coli* [40], Lee et al. have proposed the following evaluation for the function $\mathbf{f(\mu^*)}$, Eq. 11 by $\mu^*<\ln 2$:

$$f\left(\mu^*\right) = \frac{4.5 \cdot 10^{10} \cdot \mu^{*4}}{\left(78 \cdot \mu^{*2} + 233\right) \cdot \left(145 \cdot \mu^* + 82.5\right) \cdot \left(27.6 + \mu^*\right)}, \ [\mathrm{h}^{-1}] \tag{15}$$

Material balances

The material balances for biomass, limiting substrate and product for a chemostat culture, consisting of plasmid-harbouring and plasmid-free cell types (and by sterile feed) are:

Plasmid-harbouring biomass:

$$\frac{dx^+}{dt} = r_{x^+} - D \cdot x^+ \tag{16}$$

Plasmid-free biomass: $$\frac{dx^-}{dt} = r_{x^-} - D \cdot x^- \quad (17)$$

Limiting substrate: $$\frac{ds}{dt} = D \cdot (s_F - s) + r_s \quad (18)$$

Recombinant product: $$\frac{dp}{dt} = r_p - D \cdot p \quad (19)$$

where **D** and $\mathbf{s_F}$ denote the dilution rate of the chemostat and the concentration of limiting substrate in the fresh nutrient medium, respectively.

Population dynamics

For description of population dynamics of plasmid-harbouring and plasmid-free cells a model proposed by Stewart & Levin [30] was used. The equations are obtained by substitution of the kinetic relations for plasmid-harbouring and plasmid-free cells (Eq. 1 and Eq. 2) into the relevant material balances of the chemostat (Eq. 16 and Eq. 17, respectively):

$$\frac{dx^+}{dt} = \mu^+ \cdot x^+ - \Theta \cdot x^+ - D \cdot x^+ \quad (20)$$

$$\frac{dx^-}{dt} = \mu^- \cdot x^- + \Theta \cdot x^+ - D \cdot x^- \quad (21)$$

The specific rate of generation of plasmid-free cells (or specific plasmid loss rate) **Θ** is defined as a product of the relative plasmid loss rate **θ** and the specific growth rate of the plasmid-harbouring cells $\boldsymbol{\mu}^+$:

$$\Theta = \mu^+ \cdot \theta \quad (22)$$

If the total cell concentration is assumed to be constant, then Eq. 20 and Eq. 21 can be combined into a single equation describing the population dynamics by segregational plasmid instability in both batch and continuous culture [43]:

$$\frac{dz}{dt} = \Delta \cdot z^2 - (\Delta + \Theta) \cdot z \tag{23}$$

z is the ratio between concentration of plasmid-harbouring cells $\mathbf{x}^+$ and total biomass concentration **x**:

$$z = \frac{x^+}{x} \tag{24}$$

The total biomass concentration is defined as

$$x = x^+ + x^- \tag{25}$$

Δ is the difference in the specific growth rate between plasmid-free and plasmid-harbouring cells:

$$\Delta = \mu^- - \mu^+ \tag{26}$$

In general, **Δ** and **Θ** are functions of genetic characteristics, cell physiology and cultivation conditions. However, under the apparent steady-state conditions (i.e. by negligible variations in the total cell concentration and in limiting substrate concentration) the values of **Δ** and **Θ** may be assumed to be nearly constant.
