# Supplementary material for: Effects of a recombinant gene expression on ColE1-like plasmid segregation in Escherichia coli
Source: BMC Biotechnol. 2011 Mar 1;11:18. doi: 10.1186/1472-6750-11-18 (PMC3061898; doi:10.1186/1472-6750-11-18)
Supplement: Additional file 2 — Results of simulation studies using the model of Lee et al.. Simulations performed by the model of Lee et al. to study growth and hIFNγ formation kinetics of E. coli cells cultivated in a chemostat at different dilution rates. [file 1472-6750-11-18-S2.PDF]

Results of simulation studies using the model of Lee et al.

A number of simulations employing the model of Lee et al. were performed to study growth and hIFNγ formation kinetics of *E. coli* cells cultivated in a chemostat at the following dilution rates: D = 0.1, 0.4, 0.42, 0.46, 0.5, 0.55 and 0.6 h^{-1} (Additional Figure 1). All parameter values necessary for the calculations were obtained by the model of Lee et al. for *E. coli* LE392 transformed with pP_1-(SD)-hIFNγ and grown under continuous cultivation conditions (Table 3 and Table 5). For all simulations the same initial conditions were used (Table 4).

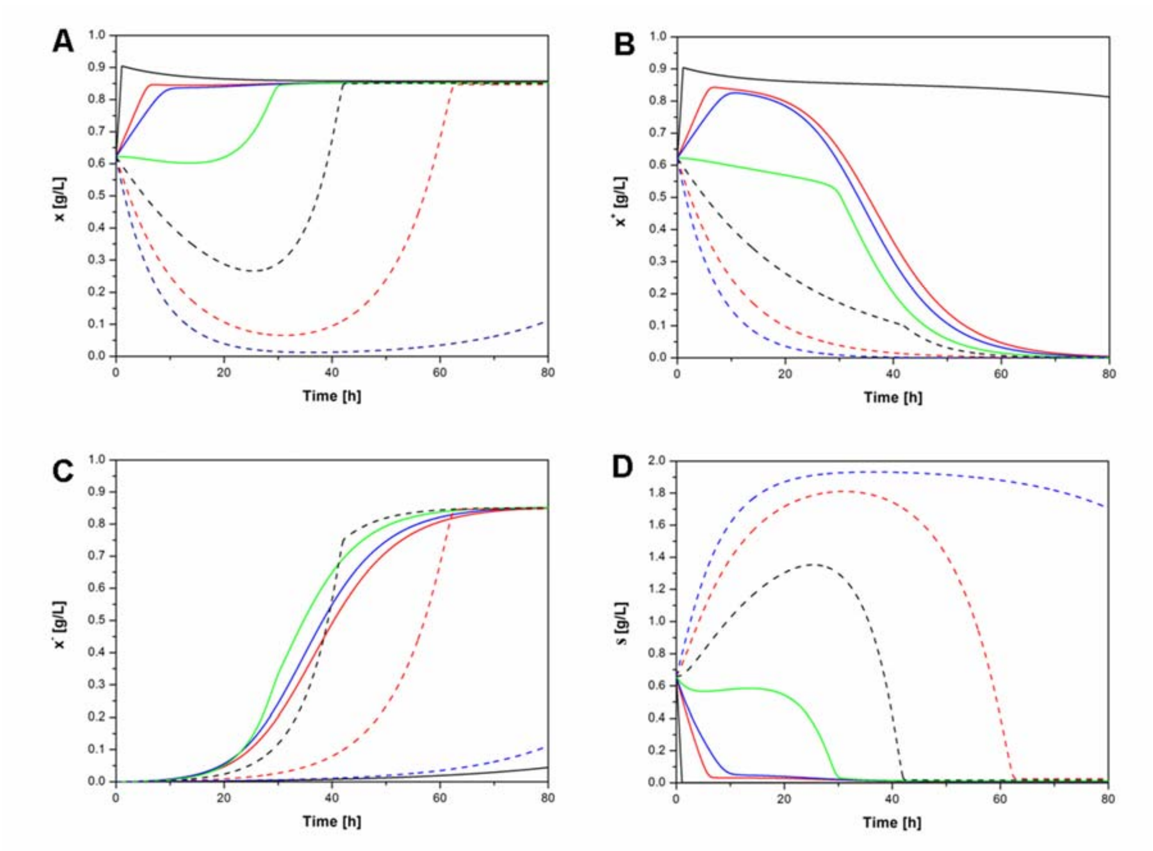

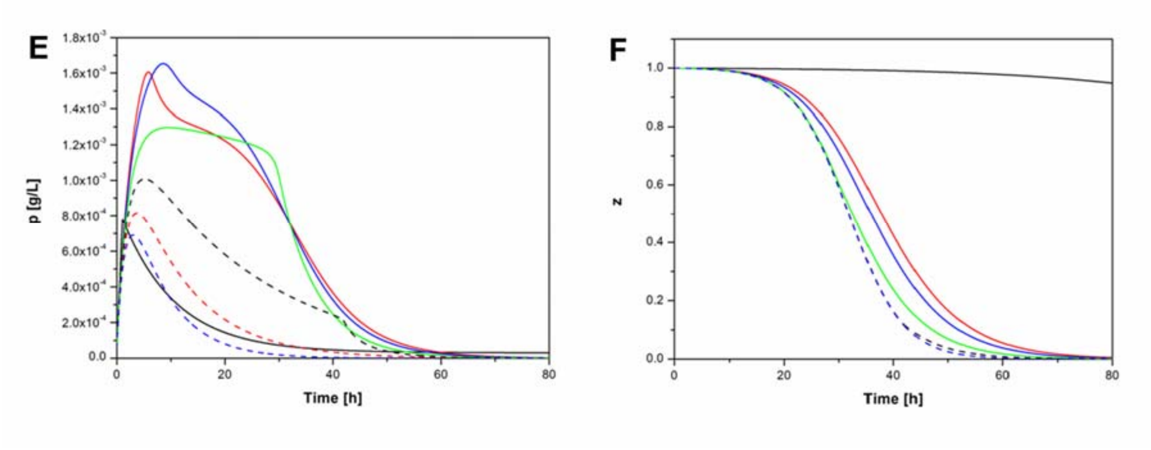

Additional Figure 1 - Simulated chemostat cultivations of *E. coli* LE392 cells carrying pP_1-(SD)-hIFNγ at different dilution rates.

Time trajectories for total cell concentration (**A**), plasmid-harbouring cell concentration (**B**), plasmid-free cell concentration (**C**), limiting substrate (**D**), recombinant product (**E**) and productive cell fraction (**F**) at different dilution rates, simulated by the model of Lee et al. Dilution rates: D = 0.1 h^{-1} (black solid line), 0.4 h^{-1} (red solid line), 0.42 h^{-1} (blue solid line), 0.46 h^{-1} (green solid line), 0.5 h^{-1} (black dashed line), 0.55 h^{-1} (red dashed line) and 0.6 h^{-1} (blue dashed line).

Additional Figure 1A presents time trajectories of the total cell concentration **x**, calculated for different dilution rates. As predicted by Lee et al. [31], at certain dilution rates (in this study higher than 0.46 h^{-1}) the total cell concentration drops and then increases, which is not observed for $\theta = 0$, i.e. for a stable plasmid copy-number (data not shown). This simulation indicates that (related to the total biomass) the concentration of the limiting substrate increases for certain dilution rates followed by a decrease (Additional Figure 1D). Additional Figure 1B, presenting the simulated time trajectories of plasmid-harbouring cells for different dilution rates, indicates that at dilution rates lower than 0.46 h^{-1} the curves are characterized by maxima, where the initial plasmid-harbouring cell concentration is

exceeded. For higher dilution rates, however, the maxima of the simulation curves correspond to the initial plasmid-harbouring cell concentration. The dependence of the maximal plasmid-harbouring cell concentration on the dilution rate is presented in Additional Figure 2.

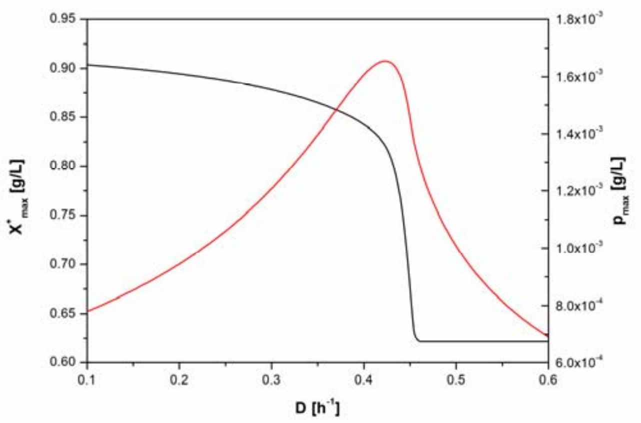

Additional Figure 2 - Simulated maximal plasmid-harbouring cell concentration and maximal concentration of recombinant protein at different dilution rates.

The maximal plasmid-harbouring cell concentration x^{+}_{max} (black line) and the maximal concentration of recombinant protein p_{max} (red line) are predicted by the model of Lee et al. for a chemostat cultivation of *E. coli* LE392 cells carrying pP1-(SD)-hIFNγ.

Additional Figure 1C shows that at dilution rates lower than 0.46 h^{-1} the cultivation time required for the plasmid-free cells to reach a certain concentration (e.g. 0.3 g/L) decreases with increasing dilution rate. However, at dilution rates higher than 0.46 h^{-1} the opposite tendency is observed. Time course of recombinant protein concentration shows sharp maxima at very low and very high dilution rates (Additional Figure 1E). Both maximum concentration of recombinant protein reached in chemostat cultivations (Additional Figure 2) and population dynamics of plasmid-

harbouring and plasmid-free cells (Additional Figure 1F) are dependent on the dilution rate. The latter is in accordance with the experimental data obtained by Chew et al. [19].
